# A Four-Dimensional Organoid System to Visualize Cancer Cell Vascular Invasion

**DOI:** 10.3390/biology9110361

**Published:** 2020-10-27

**Authors:** Kiminori Yanagisawa, Masamitsu Konno, Hao Liu, Shinji Irie, Tsunekazu Mizushima, Masaki Mori, Yuichiro Doki, Hidetoshi Eguchi, Michiya Matsusaki, Hideshi Ishii

**Affiliations:** 1 Department of Gastroenterological Surgery, Graduate School of Medicine, Osaka University, Suita, Osaka 565-0871, Japan; kyanagisawa@gesurg.med.osaka-u.ac.jp (K.Y.); mkonno@cfs.med.osaka-u.ac.jp (M.K.); tmizushima@gesurg.med.osaka-u.ac.jp (T.M.); m_mori@surg2.med.kyushu-u.ac.jp (M.M.); ydoki@gesurg.med.osaka-u.ac.jp (Y.D.); heguchi@gesurg.med.osaka-u.ac.jp (H.E.); 2 Center of Molecular Innovation and Translational Research, Graduate School of Medicine, Osaka University, Suita, Osaka 565-0871, Japan; 3 Department of Applied Chemistry, Graduate School of Engineering, Osaka University, Suita, Osaka 565-0871, Japan; h-Liu@chem.eng.osaka-u.ac.jp; 4 Joint Research Laboratory (TOPPAN) for Advanced Cell Regulatory Chemistry, Graduated School of Engineering, Osaka University, Suita, Osaka 565-0871, Japan; shinji.irie@toppan.co.jp; 5 Department of Surgery and Science, Graduate School of Medical Sciences, Kyushu University, Fukuoka 819-0395, Japan

**Keywords:** vascular organoid, vascular invasion, mimic model, 4D culture, exosome, tight junction

## Abstract

**Simple Summary:**

Using vascular organoid culture with collagen microfiber, we have established a method for culturing organoids that recapitulates the vascular invasion process of cancer cells. This culture model made it possible to four-dimensionally evaluate the dynamics of cancer cells infiltrating into blood vessels.

**Abstract:**

Vascular invasion of cancer is a critical step in cancer progression, but no drug has been developed to inhibit vascular invasion. To achieve the eradication of cancer metastasis, elucidation of the mechanism for vascular invasion and the development of innovative treatment methods are required. Here, a simple and reproducible vascular invasion model is established using a vascular organoid culture in a fibrin gel with collagen microfibers. Using this model, it was possible to observe and evaluate the cell dynamics and histological positional relationship of invasive cancer cells in four dimensions. Cancer-derived exosomes promoted the vascular invasion of cancer cells and loosened tight junctions in the vascular endothelium. As a new evaluation method, research using this vascular invasion mimic model will be advanced, and applications to the evaluation of the vascular invasion suppression effect of a drug are expected.

## 1. Introduction

Metastasis is a complex, multistep process that begins with cancer cells within the primary tumor that gain the ability to invade through the basement membrane that separates the epithelium from the underlying stroma [1]. Vascular invasion of cancer cells is an essential step in the mechanism of tumor progression that occurs during metastasis formation, and it is involved in the appearance of circulating tumor cells [2]. Numerous studies have reported the mechanism of angiogenesis, in which antivascular endothelial growth factor antibodies are used to inhibit tumor angiogenesis in animal experiments and clinical trials [3,4,5]. However, the mechanism of how cancer cells invade the vasculature vessel lumen is not understood [6,7]. When vascular invasion occurs, intercellular crosstalk between blood vessels is orchestrated with cancer cells [8]. Generally, cancer tissues are composed of complex tissue components, such as cancer-activated fibroblasts, infiltrating lymphocytes, and extracellular matrices, in addition to cancer cells [9,10]. These components contribute to the formation of a tumor microenvironment, which leads to the progression of tumors [11]. In addition, cancer-derived exosomes, a fraction of extracellular vesicles, play an important role in the formation of the tumor microenvironment, where exosomes have been reported to contribute to cancer cell proliferation, angiogenesis, tumor immunosuppression, and drug resistance [12,13]. Thus, although two-dimensional (2D) monolayer cell culture has been the gold standard in previous cancer research, cancer cells grow in the formation of a three-dimensional structure in cooperation with the tumor microenvironment [14]. Two-dimensional culture does not reflect the tumor microenvironment as an experimental model, though it is useful for studying cell-to-cell crosstalk, an essential phenomenon in tumor biology [15,16]. In vivo experiments are often used in cancer research to evaluate malignancy or antitumor effects, although it is a relatively tough experiment and has a different mechanism from humans [17]. Thus, it is difficult to observe cancer cell behavior and dynamics in detail at single-cell levels. Furthermore, in vivo experiments involve undesired problems, such as institutions, costs, and the bioethics of performing animal experiments [17]. Based on the abovementioned background, previous studies in cancer research have reported three-dimensional (3D) cancer culture methods [18] or drug discovery [19]. However, there are only a few reports on the development of an experimental model that reproduces the vascular invasion of tumors; in such reports, only limited information is gained by evaluating the vascular invasion ability of cancer cells [20,21,22,23]. Although there are a few simple and excellent vascular invasive mimic models with high reproducibility, and experiments have reported the reproduction of cancer cell invasion into capillaries utilizing a microfluidic device system [1,24], the microfluidic system is a special device that cannot be performed as an experiment in a laboratory without the system. As mentioned above, exosomes have a great influence on the formation of the tumor microenvironment. Exosomes are attracting attention in terms of cell-to-cell communication as they carry lipids, proteins, and RNA, leading to the rapid expansion of exosomes as diagnostics, treatments, and biomarkers [25,26,27]. Exosomes released by cancer cells contain many kinds of molecules that are related to angiogenesis, escaping the immune system and creating premetastatic niches that are suitable for cancer cell growth and the promotion of cancer progression [28].

To overcome these problems, useful models that reproduce the tumor microenvironment in 3D culture were developed, especially showing the relationship between cancer and blood vessels. Cell culture methods using collagen as a scaffold for tissue engineering have been established. By culturing vascular endothelial cells using a fibrin gel, it was possible to construct a vascular network with vessel lumens. By applying this technology, a cancer vascular invasion model was constructed, which can be easily created, mimics in-vivo vasculature, and thus clarifies the role of cancer-derived exosomes in terms of the mechanism of cancer cell invasion into the vascular lumen. In this study, a 3D tissue model is established, in which cancer cells invade blood vessels in the fibrin gel with collagen microfibers (CMFs), resulting in the discovery that cancer-derived exosomes weaken tight junctions in the vascular endothelium.

## 2. Results

### 2.1. Construction of Vascular Organoids in a Fibrin Gel

**Figure 1:**
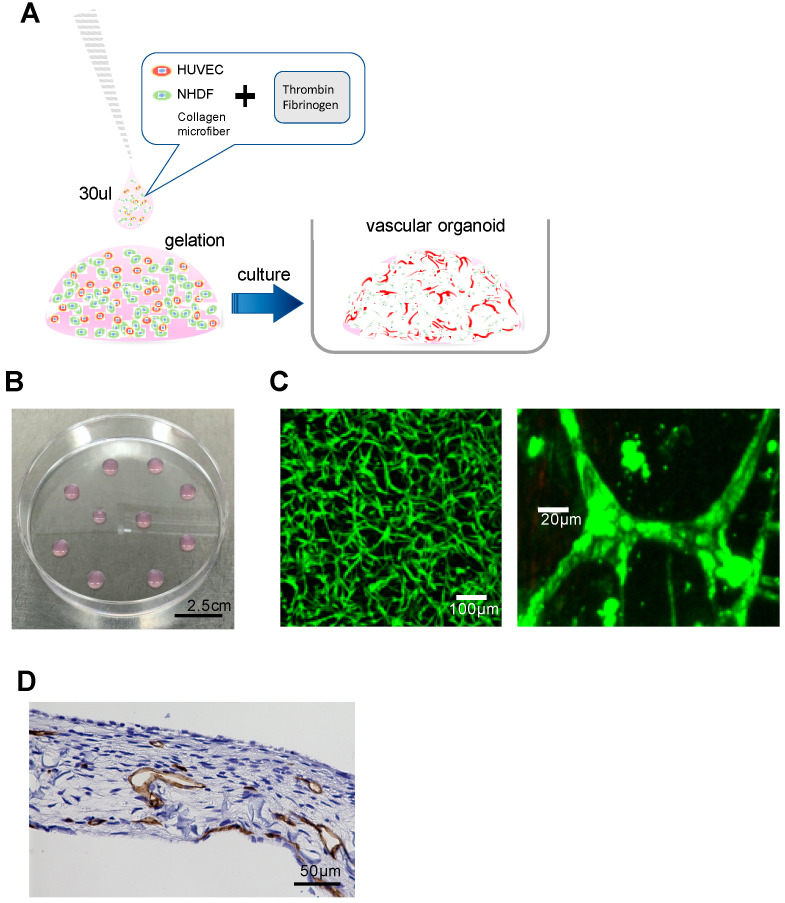
Formation of a vascular network by human umbilical vein endothelial cells (HUVECs) in a fibrin gel with collagen microfibers. (**A**) Schematic diagram of the vascular organic culture method. HUVECs and normal human dermal fibroblast cells (NHDFs) were cultured in a fibrin gel. (**B**) Appearance of the fibrin gel on a 10-cm plastic dish. Scale bar, 2.5 cm. (**C**) Confocal fluorescence microscopy image of GFP-HUVEC vascular organoids on day 3 of culture. (**D**) Immunohistological staining of vascular organoids. The first antibody is anti-CD31 (brown). These vessels formed lumens. Scale bar, 50 µm.

A culture method was constructed that easily observes vascular organoids with developed vascular networks and lumen formation. Human umbilical vein endothelial cells (HUVECs) were cultured in a fibrin gel formed by an enzymatic reaction between fibrinogen and thrombin, containing CMFs as the extracellular matrix (Figure 1A). HUVECs labeled with GFP (HUVEC-GFP) formed a vascular network in the fibrin gel. Blood vessels were approximately 20 μm in diameter (Figure 1B,C). Immunohistochemical staining using an anti-CD31 antibody revealed that the HUVECs formed lumens composed of vascular endothelial cells in the gel (Figure 1D). Confocal laser scanning microscopy (CLSM) confirmed the progress of the vascular network in a time-lapse series every hour during the culture period of 24–72 h (see Video S1).

### 2.2. Establishment and Four-Dimensional (4D) Evaluation of the Model of Cancer Cell Invasion into the Vascular Lumen

**Figure 2:**
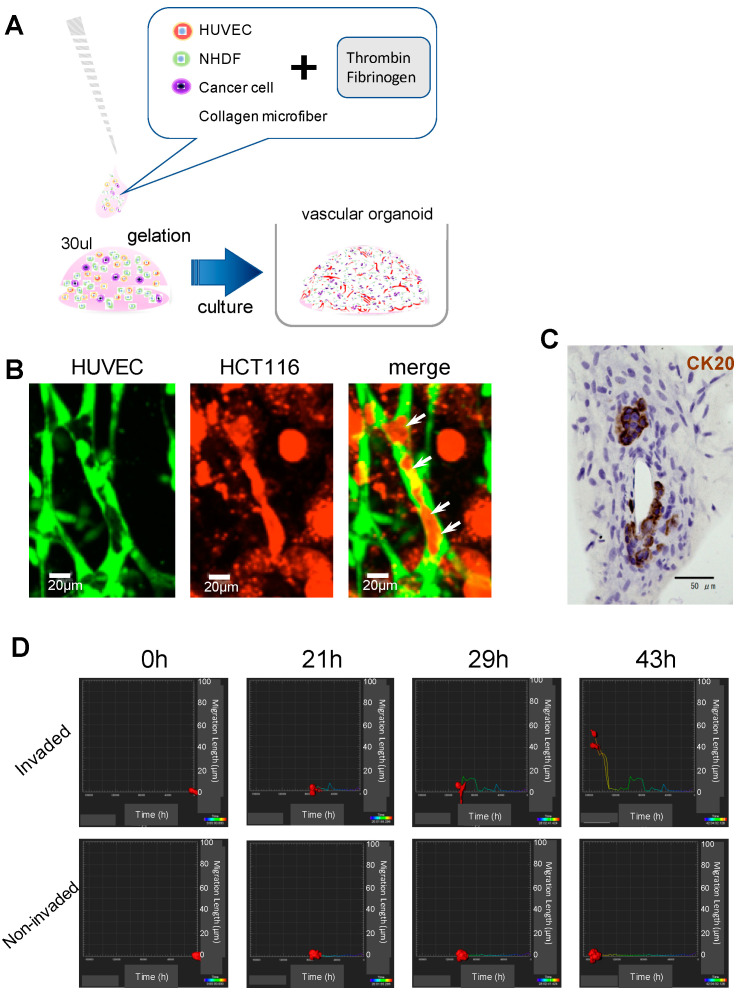
Establishment and four-dimensional evaluation of cancer cell invasion into the vascular lumen. (**A**) Schematic diagram of the culture method. Human umbilical vein endothelial cells (HUVECs), normal human dermal fibroblast cells (NHDFs), and cancer cells were cultured in a fibrin gel. (**B**) As observed by confocal laser scanning microscopy, cancer cells are contained in the blood vessel region. Each cell is labeled with a cell tracker. The arrows indicate invaded cancer cells. Scale bar, 20 µm. (**C**) Immunohistochemical staining of vascular organoids. The first antibody is anti-CK20 (brown), which binds HT29 cancer cells. These cancer cells have invaded the vascular lumen. Scale bar, 50 µm. (**D**) The migration length on the vertical axis of cancer cells within the fibrin gel at the observation period of 48 h. Two types of cells, extravascular cells and infiltrating cells, are compared. The cells in the graph show morphological changes with time on the horizontal axis. The time axis progresses from right to left.

Next, a cancer cell invasion model was created using vascular organoid techniques in a fibrin gel with CMF. First, to examine whether vascular cancer cell invasion into the vascular lumen occurred, two types of gels were used: those containing vascular organoids and those containing cancer cells. However, the cancer cells were not confirmed to have invaded the gap between the gels (see Figure S1A), suggesting that the distance between each blood vessel and cancer cell was too large to induce cancer cell invasion into vessels. Thus, a coculture of cancer cells, normal human dermal fibroblast cells (NHDFs), HUVECs, as well as CMF plus thrombin and fibrinogen, was devised, which formed the fibrin network in the gel. A large number of cancer cells (HCT116), labeled with red CMTPX dye, were deformed along the luminal side in the blood vessel, where cells were labeled with green CMFDA dye and a part of the cell body was entering the blood capillary (Figure 2A,B). To exclude any possibility that cancer cells were attached randomly to the blood vessel wall, the samples were observed by confocal microscopy, which allowed 3D analysis. HT29 cells marked in red were located inside the area of the capillaries labeled in green (see Video S2A). In sliced samples, immunohistochemical analysis, with the combination of fluorescent immunostaining, clearly confirmed that cancer cells invaded the lumen (Figure 2C and Figure S2A). On every slide that was observed in the histological examination, there was no sign of fibroblast invasion into the lumen (data not shown). Although the same cells were cocultured on a dish (2D culture), no vascular network formation or cancer cell invasion was observed (see Figure S1B). To determine whether HUVECs might be involved in the growth of HT29 cells in the fibrin gel, the growth of a single culture of HT29 and the coculture was compared; HUVECs did not affect the proliferation of HT29 cells (see Figure S1D). Next, the 4D culture in the fibrin gel was observed using CLSM in a time-lapse series every hour from 48–96 h after culture. Multiple cancer cells moved into the blood vessel regions (see Video S2B). To observe the detailed mechanism of invasive cell dynamics, time-lapse photography was performed using CLSM (Fluoview FV10i, Olympus) with a 60× objective lens every hour during a culture period of 24–72 h. After cancer cell clusters adhered to the vessel capillaries in the gel, a part of the cancer cell clusters invaded and separated into the capillaries. Additionally, it was confirmed that the cancer cell clusters moved into vessel capillaries, along with proliferation and division characteristics (see Video S3A). By observing the same region from a different angle, it was confirmed that cancer cells were moving inside the blood vessels but not outside the vessel capillaries (see Video S3B). The observation of the sample revealed that there was a difference in cell kinetics between cancer cells invading the capillary and cells distant from the capillary; the migration distance of the cells in the gel was measured using Imaris software (see Video S3C). Although cancer cells outside of the blood vessels remained in the fibrin gel without moving around, the cells that had completely entered the vessel capillary continued to extend their distance after detachment from the cancer cell cluster (Figure 2D and Figure S1E, Video S3D ).

### 2.3. Evaluation by 3D Construction

**Figure 3:**
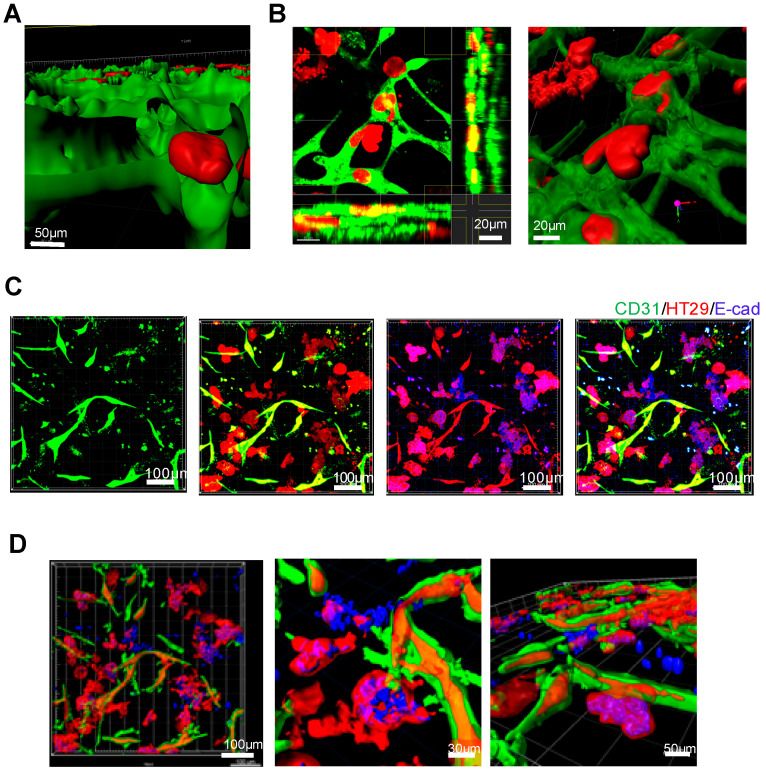
3D evaluation of the cancer vascular invasion model. (**A**) The sliced digital 3D image confirms the presence of cancer cells in the blood vessel lumen. Scale bar, 50 µm. (**B**) 3D reconstructed image with Imaris software. Cancer cells in the process of vascular invasion have undergone morphological changes. In the horizontal section image, cancer cells are contained in the blood vessel layer. Scale bar, 20 µm. (**C**,**D**) Expression evaluation of E-cadherin by fluorescent immunostaining. The blue or purple area shows E-cadherin binding. These images have been captured with confocal laser scanning microscopy and constructed with Imaris software. Scale bar, 100 µm. (**D**) The digital 3D image of E-cadherin expression in the vascular invasion model. These images reveal E-cadherin expression in different regions of the sample.

To study the detailed mechanism of the 3D positional relationship, as well as the cell morphology of cancer cells, confocal microscopic observation was performed with fluorescence signal data using Imaris software, which was analyzed by 3D image reconstruction to observe the composition of capillary lumens. The data indicated that the blood vessels induced luminal formation, and cancer cells had clearly entered the lumen (Figure 3A). Cancer cells entering the vessel capillary showed morphological changes, that is, the induction of a spindle-shape formation, reminiscent of the epithelial-to-mesenchymal transition (EMT) phenotype (Figure 3B). Given that the occurrence of EMT in capillaries was hypothesized, EMT of the corresponding sample was studied by fluorescent immunostaining with an anti-E-cadherin antibody. The expression of E-cadherin was reduced in cancer cells within vessel capillaries compared to cancer cells outside vessel capillaries, suggesting that cancer cells induced EMT when entering the vessel capillary (Figure 3C,D).

### 2.4. Involvement of Vascular Endothelium Exosomes in Vascular Invasion of Colorectal Cancer (CRC)

**Figure 4:**
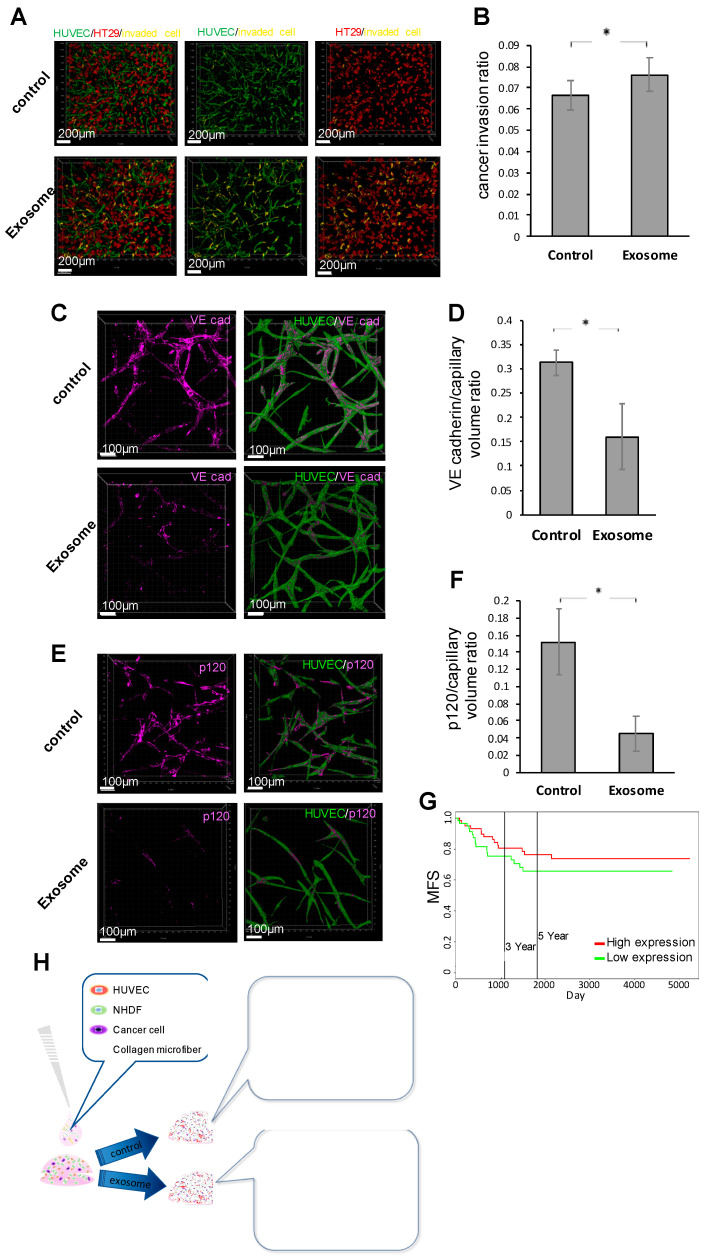
Function of cancer-derived exosomes in cancer vascular invasion. (**A**,**B**) Vascular invasion assay in a fibrin gel with collagen microfibers. (**A**) The yellow region shows the cancer cells in the vascular lumen. Scale bar, 200 µm. (**B**) The ratio of the volume of invasive cancer cells to the total volume of cancer was calculated and compared for the presence or absence of exosomes (*n* = 9, * *p* < 0.05). *p*-values were calculated using a two-tailed Student’s *t*-test. (**C**,**D**) Evaluation of changes in VE-cadherin expression in vascular organoids, with and without exosome exposure. (**C**) Fluorescent immunostaining. The purple region indicates VE-cadherin expression present in vascular organoids. Scale bar, 100 µm. (**D**) The ratio of VE-cadherin signal volume to vascular volume was calculated and compared for the presence or absence of exosomes (*n* = 9, * *p* < 0.05). *p*-values were calculated using a two-tailed Student’s *t*-test. (**E**) Fluorescent immunostaining. The purple region indicates the p120 expression present in vascular organoids. Scale bar, 100 µm. (**F**) Ratio of p120 signal volume to vessel volume was calculated and compared (*n* = 9, * *p* < 0.05). *p*-values were calculated using a two-tailed Student’s *t*-test. (**G**) Kaplan–Meier curve for metastasis-free survival (MFS) according to p120 expression in colorectal cancer, as shown by clinical database analysis using PROGgeneV2. HR: 0.14 (0.02–0.99), *p* < 0.05. (**H**) Outline and schema of results. The culture method allows observation of the vascular infiltration dynamics of cancer cells. Cancer-derived exosomes increase vascular permeability and increase cancer cell infiltration.

To determine how exosomes derived from CRC cells affect vascular endothelial cells during the invasion of tumor cells into the vascular lumen, exosomes were collected by ultracentrifugation from the culture medium of KM12-SM cells, a highly metastatic colon cancer cell line of humans [29]. The size of the collected microvesicles was measured using NanoSight (NS-10, Malvern, UK), and it was confirmed that each vesicle size was compatible with exosomes (see Figure S2A). Exosomes were labeled with PKH67 dye and were incorporated into the cytoplasm of HUVEC cells in the coculture of HUVECs and exosomes (see Figure S2B). Although the effect of exosomes exposed to the formation of vascular networks was studied, no significant difference was observed in vessel volume in the fibrin gel (see Figure S2C). Then, exosome-exposed HUVECs were cocultured with cancer cells in the gel, and the degree of invasion by the cancer cells was studied. The proportion of cancer cells that invaded vessels was significantly higher in the exosome-exposed group than in the nonexposed control group (Figure 4A,B). To study the enhancement mechanism of cancer cell invasion into the vascular lumen by exosomes, immunohistochemical analysis of p120 and VE-cadherin was performed in the vessels, which are reportedly tight-junction-related proteins in the vascular endothelium [30,31]. To study the involvement of cancer cells in the expression of these proteins, vascular organoids were cultured using HUVECs exposed to exosomes, but the HUVECs did not contain cancer cells in the gel; then, the expression of each protein was studied by fluorescence staining and signal volume calculation. The expression of VE-cadherin in the vascular endothelium was significantly lower in the exosome-exposed group than in the nonexposed control group (Figure 4C,D). Similarly, the expression of the p120 protein in the vascular endothelium was significantly lower in the exosome-exposed group than in the nonexposed control group (Figure 4E,F). These results suggested that cancer-derived exosomes reduced vascular endothelium tight junctions and increased the vascular permeability of cancer cells. Clinical open database analysis, using the PROGgeneV2 database and dataset GSE28814, confirmed that metastasis-free survival (MFS) was significantly higher in the p120 high-expression group than that in the low-expression group (Figure 4G). This suggested that MFS might be affected by exosomes derived from cancer cells, following the alteration of tight junctions.

## 3. Discussion

CRC is the third most common cancer in the world and the fourth most common cause of cancer death, and it is expected to increase in the future [32,33]. Prognosis has improved due to developments in surgical techniques and chemotherapeutic approaches against CRC [34], although stage IV CRC patients with recurrence and metastasis have a 5-year survival rate of 20–30% and a poor prognosis [34,35]. Therefore, further treatment development is desired. Generally, cancer metastasis occurs in the advanced stages of tumors, of which aggressive behaviors are involved in the prognosis of cancer patients. To improve the outcome of colorectal cancer, further studies of the mechanism of cancer metastasis and invasion, as well as the development of new therapeutic approaches, are necessary. To the best of our knowledge, no drugs that directly inhibit the invasion of blood vessels have been developed for clinical settings. Given that it is difficult to achieve complete eradication of cancer metastases with standard chemoradiation treatments, the development of innovative methods to inhibit cancer metastasis, especially controlling the invasion of cancer cells into the vascular lumen, is required. Using the present invasion mimic model of cancer cells into the vascular lumen to conduct research, it will be possible to elucidate the mechanism of cancer cell invasion in detail, which can lead to the development of inhibitors for invasion.

Reportedly, 3D culture has cell–cell interaction and maintains a cell function that is closer to that of a living body compared to 2D monolayer culture [15]. In addition, procedures in 3D culture are easier to perform, and 3D culture has increased reproducibility compared to in-vivo animal experiments, showing the benefit of 3D culture. Considering the emerging surveillance of compliance rules for ethical issues in animal experiments [17], developing 3D organoid research would be more advantageous for cancer studies than other methods. Beyond the 3D structure, this method can be further applied to the novel mimic model that enables 4D evaluation of vascular invasion using time-lapse videos with a time axis. In the vascular invasion mimic model, it is useful to evaluate cell dynamics and functions that are difficult to evaluate at the cellular level in in-vivo experiments; eventually, the involvement of the p120 protein and the EMT was observed (Figure 4H). In hepatocellular carcinoma, cancer-derived exosomes increase vascular permeability as an effect on the tumor microenvironment [35,36]. These results show the same phenomenon, which suggests that this is a reliable model for vascular invasion. The present mimic model enables the elucidation of the mechanism of vascular invasion and the application of drug tests on the vascular invasion.

Although previous studies have reported modeling invasion of cancer cells into the vascular lumen, those studies used endothelial cells in a monolayer manner, in which cancer cells pass through [37]; therefore, they are not suitable for the study of vascular infiltration of cancer cells utilizing vascular organoids. In this regard, in vitro pathological mimic models of the tumor microenvironment are easily constructed, highly reproducible, and easy to evaluate, compared to in-vivo experiments and microfluidic devices. Thus, in the present study, a new, important platform for promoting invasion research of cancer cells is created. In vivo imaging does not allow the study of detailed mechanisms of tissues in a real-time manner. In the case of 3D evaluation of biological tissues with a fluorescence optical microscope, it is essential to make the tissue transparent for observations; however, transparency requires a lot of time and labor. In sharp contrast, the 3D culture in the present study was performed in a fibrin gel with collagen microfibers. The sample was studied without clearing tissues, and the cell dynamics and histological positional relationship was studied in real-time. During cancer cell progression, cancer cells undergo various changes in their invasion, including EMT, to become circulating tumor cells. To study this, it is necessary to understand the dynamics of such cells and to detect genetic changes in each situation. To achieve this, time-lapse observation of cell dynamics is an important observation method.

The present mimic model has some limitations: the vessel capillary has a monolayer structure and lacks the backing of the basement membrane and pericytes. Therefore, the possibility that the barrier mechanism against infiltration may be weaker than the original vasculature must be considered. In the vascular invasion model, there is also no fluid flow in the vessel, and the mechanism of cancer cell movement in the vessel is unknown. Although E-cadherin expression was evaluated, it is unclear when cancer cells decrease the expression of E-cadherin during the vascular invasion. Nevertheless, this mimic model has merits. A vascular organoid and invasion model was easily made, with high reproducibility, and was used to clearly observe the samples in detail.

## 4. Materials and Methods

### 4.1. Cell Lines and Cell Culture

Human colorectal cancer (CRC) cell lines KM12-SM and HCT116 were purchased from the American Type Culture Collection (Manassas, VA, USA). KM12-SM was established from liver metastasis spices that were generated by KM12 injection in nude mouse [29]. KM12 was established from primary human colorectal carcinoma [29]. HCT116 was established from human colorectal carcinoma. The normal human dermal fibroblast cell (NHDF), human umbilical vein endothelial cell (HUVEC), and endothelial growth medium (EGM-2MV) were purchased from Lonza (Basel, Switzerland). GFP-expressed HUVEC was purchased from Angio-Proteomie (Boston, MA, USA). RFP-expressed HT29 were purchased from Anticancer (San Diego, CA, USA). HT29 is a human colorectal adenocarcinoma cell line. KM12-SM, HCT116, NHDF and HT29-RFP were cultured in Dulbecco modified Eagles’s medium (DMEM) High-Glucose (Nacalai tesque, 08458, Kyoto, Japan) with antibiotics (Nacalai tesque, 02894, Kyoto, Japan), supplemented with 10% FBS at 37 °C and 5% CO_2_ in a humidified incubator. HUVEC and HUVEC-GFP were cultured in endothelial growth medium (EGM-2MV, Lonza CC4147, Basel, Switzerland) supplemented with 10% FBS at 37 °C at 5% CO_2_ in a humidified incubator.

### 4.2. Vascular Organoids in a Fibrin Gel with CMF

Collagen type 1 sponges from pigs were kindly provided by Nippon Ham (Osaka, Japan). The CMF-200 µm was fabricated from a collagen type 1 sponge after dehydration condensation, based on a previous study [38]. The collagen type 1 sponge was dehydrothermally treated by drying with heat under a vacuum at 200 °C for 24 h for crosslinking. The crosslinked collagen sponge was mixed with 10× phosphate-buffered saline (PBS) solution at a concentration of 10 mg/mL (pH = 7.4, 25 °C), and a 6-min homogenization (30,000 rpm) was performed using a Violamo VH-10 homogenizer (S10N-10G), with a probe 10 mm in diameter and 115 mm in length. The CMF-20 µm suspension was obtained with further ultrasonication (ultrasonic processor VC50, 50 W, 20 kHz) in an ice bath for 100 cycles (1 cycle included 20 s ultrasonication and 10 s cooling). The solution was transferred to a glass recipient after filtration (40 µm filter, microsyringe 25 mm filter holder, Merck), and the filtrate was freeze-dried for 48 h (freeze dryer FDU-2200, Eyela Co., Tokyo, Japan). The obtained CMF-20 µm was kept in a desiccator at room temperature (RT). For the preparation of 3D capillary tissue, NHDFs and HUVECs were trypsinized (5 min, 37 °C) and collected by centrifugation (5 min, 1000 rpm, RT). The culture solution used in the following operation was a mixture of EGM-2 and DMEM (FBS free, 1% antibiotics) in equivalent amounts. CMF-20 µm (0.15 mg), thrombin (0.15 Unit), and cells (1 × 10^5^ NHDFs and 5 × 10^4^ HUVECs) were mixed with 20 µL medium, and then 0.15 mg of fibrinogen was dissolved in 10 µL of medium at 37 °C for 30 min to prepare the fibrinogen solution; two types of solutions were mixed in a tube. The completed mixture (30 µL) was quickly dropped onto the culture dish or glass-bottom dish. In the vascular invasion model, 1 × 10^4^ cancer cells were added and mixed with NHDFs and HUVECs, as described above. After 30 min of gelation at 37 °C, the gel samples were further cultured at 37 °C and 5% CO_2_ in a humidified incubator.

### 4.3. Immunohistochemical Staining

The expression of CD31 was assessed by immunohistochemical staining of formalin-fixed and paraffin-embedded vascular organoids in a fibrin gel with CMF-20 µm. To liberate antibody-binding sites using L.A.B. solution (Polysciences Inc., Warrington, PA, USA) and to block endogenous peroxidase activity, blocking was performed for 20 min at room temperature using a VECTASTAIN Elite ABC kit (mouse IgG; #PK-6102; Vector Laboratories, Burlingame, CA, USA). Then, 3.5-µm thick sections were incubated overnight at 4 °C with the mouse monoclonal anti-CD31 antibody (dilution, 1:200; Wako, M0823, Osaka, Japan). Hematoxylin was used for nuclear staining for 1 min. Dehydration was performed using a graded ethanol series of 60%, 70%, 80%, 90%, and 95% ethanol for 1 min each, 100% ethanol for 2 min, twice, and xylene for 5 min, 3 times. Images were captured using a BZ-710 All-in-One Fluorescence Microscope (KEYENCE Corporation, Osaka, Japan).

### 4.4. Cell Tracker Labeling

HCT116 and HUVEC cells were labeled with either red CMTPX dye or green CMFDA dye (Carlsbad, CA, USA), according to the manufacturer’s protocol. Confluent cells (~80% confluent) on a 10-cm dish were washed with PBS and then incubated with DMEM containing 1/1000 Cell Tracker dye at 37 °C for 30 min and kept in the dark. After incubation, the DMEM was removed, and cells were washed with PBS for subsequent experiments.

### 4.5. Fluorescence Imaging and Histological Analysis of 3D Capillary Tissue

Nuclei were stained with Hoechst 33324 (H3570, Thermo Fisher Scientific, Waltham, MA, USA), and HUVECs were detected with anti-CD31 antibody. Briefly, the fibrin gel tissues were fixed in 4% paraformaldehyde (30 min, RT) and permeabilized with 0.02% Triton X-100 (T8787, Sigma-Aldrich, St. Louis, MO, USA) for 15 min. The tissues were blocked with 1 wt% bovine serum albumin (BSA, A3294, Sigma-Aldrich, St. Louis, MO, USA) for 2 h and then incubated with the primary antibodies, anti-CD31 rabbit antibody (ab28364, Abcam, Cambridge, UK), anti-E-cadherin mouse antibody (sc-8426, Santa Cruz, TX, USA), anti-VE cadherin mouse antibody (sc-9989, Santa Cruz, TX, USA), and anti-p120 mouse antibody (sc-23873, Santa Cruz, Texas, USA), diluted 1/200 in BSA at 4 °C overnight. After washing the tissues with PBS, secondary antibodies (goat-anti mouse, Alexa Fluor 647, goat-anti rabbit, Alexa Fluor 488, 1:200 dilution in 1% BSA) were added. After rinsing with PBS, the 3D tissue was finally observed using CLSM (Confocal Quantitative Image Cytometer CQ1, Yokogawa, Japan; CLSM Fluoview FV3000, Olympus, Japan and CLSM, Fluoview FV10i, Olympus, Japan). Imaris software (ver. 9.2.1, Oxford Instruments, Pleasanton, CA, USA) reconstructed 3D images from CLSM data and calculated the volume of capillary blood, cancer cells, VE-cadherin, and p120.

### 4.6. Exosome Isolation and Treatment

Exosomes were purified from CRC-derived conditioned medium (CM) by ultracentrifugation. CRC cell lines were cultured in DMEM supplemented with 3% exosome-depleted FBS (Exo-FBS-50A-1, SBI, Fremont, CA, USA). CM was collected after 48 h of cell culture and centrifuged at 500× *g* for 10 min at 4 °C, followed by centrifugation at 2000× *g* for 10 min at 4 °C. The supernatants were passed through a 0.22 µm filter (8020, IWAKI, Shizuoka, Japan) and ultracentrifuged at 174,900× *g* for 84 min at 4 °C. The exosomal pellets were washed with PBS, followed by a second ultracentrifugation at 174,900× *g* for 84 min at 4 °C, and then resuspended in PBS. An Optima WE-90 (Beckman, Brea, CA, USA) with SW32Ti as a swing rotor was used for ultracentrifugation. The amount of exosomes was measured as protein using the Qubit3.0 fluorometer (Thermo Fisher Scientific, Waltham, MA, USA). When exosomes were exposed to cells, cell culture was performed by adjusting the medium so that the exosome concentration was 20 µg/mL.

To examine the cellular internalization of exosomes, exosomes were labeled with PKH67GL (Sigma-Aldrich, St. Louis, MO, USA), added to HUVECs at 80% confluence and incubated for 24 h before imaging under CLSM (FV3000, Olympus, Tokyo, Japan). The nucleus was labeled with Hoechst 33324.

### 4.7. Ethic Committee Approval and Code

The experiments were approved by experimental ethical committee under code number 4305 in Osaka University, Japan.

## 5. Conclusions

As a new evaluation method, the constructed 3D tissue model will contribute to medical cancer care by suppressing the invasion of cancer cells and helping to control metastasis and cancer recurrence.

## Supplementary Materials

The following are available online at https://www.mdpi.com/2079-7737/9/11/361/s1. Figure S1: The interaction between cancer cells and vascular endothelial cells, Figure S2: Effects of cancer-derived exosomes on vascular endothelial cells, Video S1: Time-lapse microscopy recordings of vascular network formation, Video S2: Three-dimensional evaluation of the positional relationship between vessels and cancer cells in vascular invasion model, Video S3: Time-lapse microscopy recordings of cancer vascular invasion with a CLSM every hour for 24 to 72 h of culture.

## References

[1] Sontheimer-Phelps A., Hassell B.A., Ingber D.E. (2019). Modelling cancer in microfluidic human organs-on-chips. Nat. Rev. Cancer.

[2] Luai R.Z., Anand S., Billingsley K.G., Bisson W.H., Cercek A., Clarke M.F., Coussens L.M., Gast C.E., Geltzeiler C.B., Hansen L. (2017). Colorectal cancer liver metastasis: Evolving paradigms and future directions. Cell. Mol. Gastroenterol. Hepatol..

[3] Ferrara N., Hillan K.J., Novotny W. (2005). Bevacizumab (Avastin), a humanized anti-VEGF monoclonal antibody for cancer therapy. Biochem. Biophys. Res. Commun..

[4] Pavlidis E.T., Pavlidis T.E. (2013). Role of bevacizumab in colorectal cancer growth and its adverse effects: A review. World J. Gastroenterol..

[5] Deok-Hoon K., Kim M.R., Jang J.H., Na H.J., Lee S. (2017). A Review of anti-angiogenic targets for monoclonal antibody cancer therapy. Int. J. Mol. Sci..

[6] Reymond N., d’Água B.B., Ridley A.J. (2013). Crossing the endothelial barrier during metastasis. Nat. Rev. Cancer.

[7] Shenoy A.K., Lu J. (2016). Cancer cells remodel themselves and vasculature to overcome the endothelial barrier. Cancer Lett..

[8] Kikuchi S., Yoshioka Y., Prieto-Vila M., Ochiya T. (2019). Involvement of extracellular vesicles in vascular-related functions in cancer progression and metastasis. Int. J. Mol. Sci..

[9] Kalluri R. (2016). The biology and function of fibroblasts in cancer. Nat. Rev. Cancer.

[10] Di Modugno F., Colosi C., Trono P., Antonacci G., Ruocco G., Nisticò P. (2019). 3D models in the new era of immune oncology: Focus on T cells, CAF and ECM. J. Exp. Clin. Cancer Res..

[11] Roma-Rodrigues C., Mendes R., Baptista P.V., Fernandes A.R. (2019). Targeting tumor microenvironment for cancer therapy. Int. J. Mol. Sci..

[12] Kahlert C., Kalluri R. (2013). Exosomes in tumor microenvironment influence cancer progression and metastasis. J. Mol. Med..

[13] Naito Y., Yoshioka Y., Yamamoto Y., Ochiya T. (2017). How cancer cells dictate their microenvironment: Present roles of extracellular vesicles. Cell. Mol. Life Sci..

[14] Hoarau-Véchot J., Rafii A., Touboul C., Pasquier J. (2018). Halfway between 2D and animal models: Are 3D cultures the ideal tool to study cancer-microenvironment interactions?. Int. J. Mol. Sci..

[15] Yamada K.M., Cukierman E. (2007). Modeling tissue morphogenesis and cancer in 3D. Cell.

[16] Blaha L., Zhang C., Cabodi M., Wong J.Y. (2017). A microfluidic platform for modeling metastatic cancer cell matrix invasion. Biofabrication.

[17] Cheluvappa R., Scowen P., Eri R. (2017). Ethics of animal research in human disease remediation, its institutional teaching; and alternatives to animal experimentation. Pharmacol. Res. Perspect..

[18] Ravi M., Ramesh A., Pattabhi A. (2017). Contributions of 3D cell cultures for cancer research. J. Cell. Physiol..

[19] Weeber F., Ooft S.N., Dijkstra K.K., Voest E.E. (2017). Tumor organoids as a pre-clinical cancer model for drug discovery. Cell Chem. Biol..

[20] Jeon J.S., Bersini S., Gilardi M., Dubini G., Charest J.L., Moretti M., Kamm R.D. (2015). Human 3d vascularized organotypic microfluidic assays to study breast cancer cell extravasation. Proc. Natl. Acad. Sci. USA.

[21] Chen M.B., Whisler J.A., Fröse J., Yu C., Shin Y., Kamm R.D. (2017). On-chip human microvasculature assay for visualization and quantification of tumor cell extravasation dynamics. Nat. Protoc..

[22] Xu Z., Li E., Guo Z., Yu R., Hao H., Xu Y., Sun Z., Li X., Lyu J., Wang Q. (2016). Design and construction of a multi-organ microfluidic chip mimicking the in vivo microenvironment of lung cancer metastasis. ACS Appl. Mater. Interfaces.

[23] Nishiguchi A., Matsusaki M., Kano M.R., Nishihara H., Okano D., Asano Y., Shimoda H., Kishimoto S., Iwai S., Akashi M. (2018). In vitro 3D blood/lymph-vascularized human stromal tissues for preclinical assays of cancer metastasis. Biomaterials.

[24] Bersini S., Moretti M. (2015). 3D functional and perfusable microvascular networks for organotypic microfluidic models. J. Mater. Sci. Mater. Med..

[25] Mathivanan S., Ji H., Simpson R.J. (2010). Exosomes: Extracellular organelles important in intercellular communication. J. Proteom..

[26] Ludwig A.K., Giebel B. (2012). Exosomes: Small vesicles participating in intercellular communication. Int. J. Biochem. Cell Biol..

[27] Meldolesi J. (2018). Exosomes and ectosomes in intercellular communication. Curr. Biol..

[28] Carolina F.R., Adem B., Silva M., Melo S.A. (2017). The biology of cancer exosomes: Insights and new perspectives. Cancer Res..

[29] Morikawa K., Walker S.M., Nakajimam M., Pathak S., Jessup J.M., Fidler I.J. (1988). Influence of organ environment on the growth, selection, and metastasis of human colon carcinoma cells in nude mice. Cancer Res..

[30] Nanes B.A., Grimsley-Myers C.M., Cadwell C.M., Robinson B.S., Lowery A.M., Vincent P.A., Mosunjac M., Früh K., Kowalczyk A.P. (2017). p120-catenin regulates VE-cadherin endocytosis and degradation induced by the Kaposi sarcoma-associated ubiquitin ligase K5. Mol. Biol. Cell.

[31] Garrett J.P., Lowery A.M., Adam A.P., Kowalczyk A.P., Vincent P.A. (2017). Regulation of endothelial barrier function by p120-catenin∙VE-cadherin interaction. Mol. Biol. Cell.

[32] Arnold M., Sierra M.S., Laversanne M., Soerjomataram I., Jemal A., Bray F. (2017). Global patterns and trends in colorectal cancer incidence and mortality. Gut.

[33] Bray F., Ferlay J., Soerjomataram I., Siegel R.L., Torre L.A., Jemal A. (2018). Global cancer statistics 2018: GLOBOCAN estimates of incidence and mortality worldwide for 36 cancers in 185 countries. CA Cancer J. Clin..

[34] Colvin H., Mizushima T., Eguchi H., Takiguchi S., Doki Y., Mori M. (2017). Gastroenterological surgery in Japan: The past, the present and the future. Ann. Gastroenterol. Surg..

[35] Yang N., Li S., Li G., Zhang S., Tang X., Ni S., Jian X., Xu C., Zhu J., Lu M. (2017). The role of extracellular vesicles in mediating progression, metastasis and potential treatment of hepatocellular carcinoma. Oncotarget.

[36] Fang J.H., Zhang Z.J., Shang L.R., Luo Y.W., Lin Y.F., Yuan Y., Zhuang S.M. (2018). Hepatoma cell-secreted exosomal microRNA-103 increases vascular permeability and promotes metastasis by targeting junction proteins. Hepatology.

[37] Flores P.A., Rincón D.G., Ruiz-García E., Echavarria R., Marchat L.A., Álvarez-Sánchez E., López-Camarillo C. (2018). Angiogenesis analysis by in vitro coculture assays in transwell chambers in ovarian cancer. Methods Mol. Biol..

[38] Gorham S.D., Light N.D., Diamond A.M., Willins M.J., Bailey A.J., Wess T.J., Leslie N.J. (1992). Effect of chemical modifications on the susceptibility of collagen to proteolysis. II. Dehydrothermal crosslinking. Int. J. Biol. Macromol..

