# Supplementary material for: A Four-Dimensional Organoid System to Visualize Cancer Cell Vascular Invasion
Source: Biology (Basel). 2020 Oct 27;9(11):361. doi: 10.3390/biology9110361 (PMC7692192; doi:10.3390/biology9110361)
Supplement: Supplementary file 1 [file biology-09-00361-s001.zip › 200827Suppl.docx]

**Four-dimensional organoid system to visualize vascular invasion of cancer cells**

Kiminori Yanagisawa^1,2^, Masamitsu Konno^3^, Hao Liu^4^, Shinji Irie^5^, Ayumu Asai^6^, Tsunekazu Mizushima^1^, Taroh Satoh^3^, Masaki Mori^7^, Yuichiro Doki^1^, Hidetoshi Eguchi^1^, Michiya Matsusaki^4^, and Hideshi Ishii^2^

**Affiliations**

^1)^Department of Gastroenterological Surgery, Graduate School of Medicine, Osaka University, Suita, Osaka, Japan.

^2)^Department of Medical Data Science, Graduate School of Medicine, Osaka University, Suita, Osaka, Japan.

^3)^Department of Frontier Science for Cancer and Chemotherapy, Graduate School of Medicine, Osaka University, Suita, Osaka, Japan.

^4)^Department of Applied Chemistry, Graduate School of Engineering, Osaka University, Suita, Osaka, Japan.

^5)^Joint Research Laboratory (TOPPAN) for Advanced Cell Regulatory Chemistry, Graduated School of Engineering, Osaka University, Suita, Osaka, Japan.

^6)^Artificial Intelligence Research Center, The Institute of Scientific and Industrial Research, Osaka University, Ibaraki, Osaka, Japan.

^7)^Department of Surgery and Science, Graduate School of Medical Sciences, Kyushu University, Fukuoka, Fukuoka, Japan.

**Supplementary Figure Legends**

**Supplementary Figure S1**

(A) The fibrin gel with CMF containing vascular organoids and cancer cells was adhered. The right figure is an enlarged image of the bonded portion. Left figure Scale bar, 1500µm. Right figure Scale bar, 80µm. (B) Fluorescent immunological double staining with sliced samples of vascular invasion organoids. (C) Observation of cell culture in 2D culture. HUVEC-GFP, HT29-RFP, and NHDF were cultured on a dish and observed under the same cell number conditions as in 3D culture. Scale bar, 100µm (D) Effect of HUVEC on the growth of cancer cells in fibrin gel with CMF. The volume of cancer cells per field of CLMS image in the gel was measured and compared. (n=12) *P* values were calculated using a two- tailed Student’s t-test. N.S., Not Significant; CMF, collagen microfiber.

**Supplementary Figure S2**

(A) Exosome detection by NanoSight LM10 analysis. (B) Exosome uptake into HUVEC. Exosomes were fluorescently labeled with PKH67. Scale bar, 10µm (C) Comparison of vascular network formation with and without exosomes. The blood vessel volume in the field of CLSM images was calculated with Imaris (n=9). *P* values were calculated using a two-tailed Student’s t-test. N.S.: Not Significant

**Supplementary Video Legends**

**Supplementary Video S1.** Time-lapse microscopy recordings of vascular network formation. Time-lapse photography with a confocal fluorescence scanning microscope (CLSM) every hour for 24 to 72 hours of culture. The environment in the culture vessel was maintained at a temperature of 37℃, a humidity of 90% and a CO2 concentration of 5%.

**Supplementary Video S2.** Three-dimensional evaluation of the positional relationship between vessels and cancer cells in vascular invasion model**.** (A)Three-dimensional rotating image of cancer vascular invasion site. Cancer cells are contained within the green blood vessel area. (B) Evaluation of cancer cells during vascular invasion. Fluorescent signal data was three-dimensionally constructed with Imaris, and the vessel lumen and invasive cancer cells were observed.

**Supplementary Video S3.** Time-lapse microscopy recordings of cancer vascular invasion with a CLSM every hour for 24 to 72 hours of culture. (A) Observation of organoid culture process with 10x objective lens. (B, C) Detailed observation using a 60x objective lens. (C) The video of vascular invasion point observed from different angles. In each observation, cancer cells migrate not outside the vessel but inside. (D) The length of migrating cancer cells within the fibrin gel in the observation period of 48 hours. Two types of cells, extravascular cells and infiltrating cells, are compared. The cells in the graph show morphological changes with time.
