# Supplementary figures and images for: A Four-Dimensional Organoid System to Visualize Cancer Cell Vascular Invasion
Source: Biology (Basel). 2020 Oct 27;9(11):361. doi: 10.3390/biology9110361 (PMC7692192; doi:10.3390/biology9110361)

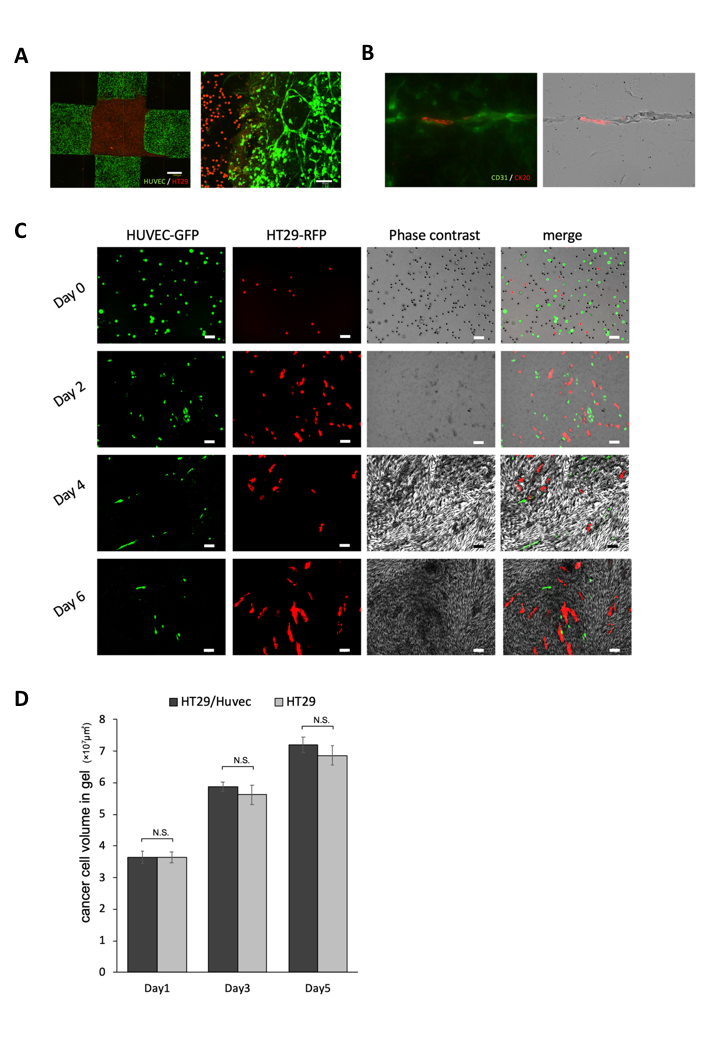

Supplement: Supplementary file 1 [file biology-09-00361-s001.zip › Supplementary Fig S1.tiff]

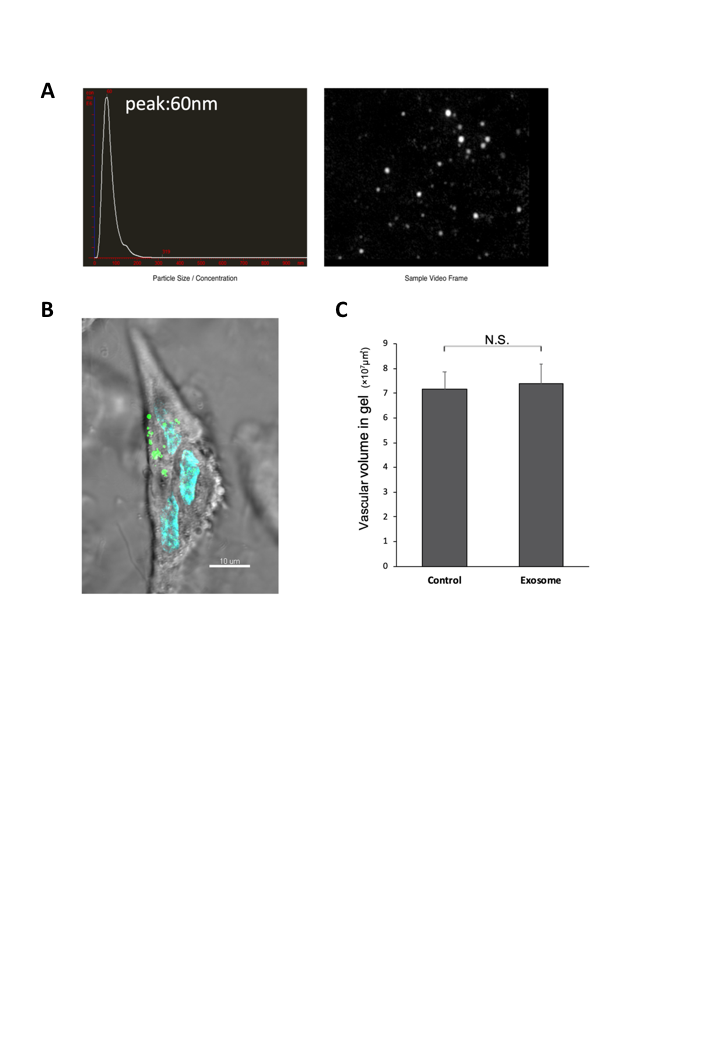

Supplement: Supplementary file 1 [file biology-09-00361-s001.zip › Supplementary Fig S2.tiff]
